# Long-Range Enhancer Associated with Chromatin Looping Allows AP-1 Regulation of the Peptidylarginine Deiminase 3 Gene in Differentiated Keratinocyte

**DOI:** 10.1371/journal.pone.0003408

**Published:** 2008-10-16

**Authors:** Stéphane Chavanas, Véronique Adoue, Marie-Claire Méchin, Shibo Ying, Sijun Dong, Hélène Duplan, Marie Charveron, Hidenari Takahara, Guy Serre, Michel Simon

**Affiliations:** 1 UMR 5165, CNRS-Toulouse III University, CHU Purpan, Toulouse, France; 2 Department of Applied Biological Resource Sciences, School of Agriculture, Ibaraki University, Ibaraki, Japan; 3 Institut de Recherche Pierre Fabre, Toulouse, France; Deutsches Krebsforschungszentrum, Germany

## Abstract

Transcription control at a distance is a critical mechanism, particularly for contiguous genes. The peptidylarginine deiminases (PADs) catalyse the conversion of protein-bound arginine into citrulline (deimination), a critical reaction in the pathophysiology of multiple sclerosis, Alzheimer's disease and rheumatoid arthritis, and in the metabolism of the major epidermal barrier protein filaggrin, a strong predisposing factor for atopic dermatitis. PADs are encoded by 5 clustered *PADI* genes (1p35-6). Unclear are the mechanisms controlling the expression of the gene *PADI*3 encoding the PAD3 isoform, a strong candidate for the deimination of filaggrin in the terminally differentiating epidermal keratinocyte. We describe the first PAD Intergenic Enhancer (PIE), an evolutionary conserved non coding segment located 86-kb from the *PADI*3 promoter. PIE is a strong enhancer of the *PADI*3 promoter in Ca2+-differentiated epidermal keratinocytes, and requires bound AP-1 factors, namely c-Jun and c-Fos. As compared to proliferative keratinocytes, calcium stimulation specifically associates with increased local DNase I hypersensitivity around PIE, and increased physical proximity of PIE and *PADI*3 as assessed by Chromosome Conformation Capture. The specific AP-1 inhibitor nordihydroguaiaretic acid suppresses the calcium-induced increase of *PADI*3 mRNA levels in keratinocytes. Our findings pave the way to the exploration of deimination control during tumorigenesis and wound healing, two conditions for which AP-1 factors are critical, and disclose that long-range transcription control has a role in the regulation of the gene *PADI*3. Since invalidation of distant regulators causes a variety of human diseases, PIE results to be a plausible candidate in association studies on deimination-related disorders or atopic disease.

## Introduction

The characterization of the genomic elements which govern the spatial and temporal tuning of gene transcription is a key issue in genomic biology, providing with a better understanding of the cell regulatory networks and the functional landscape of the genome [1]. Notably, transcription control at a distance is a critical mechanism, providing with cell-type specificity of transcription or coordinate expression of clustered genes [2]. The 5 genes (*PADI*) for the peptidylarginine deiminases (PADs) are clustered on chromosome 1p35-36 (Genbank AJ549502) [3]. PADs catalyse the conversion of protein-bound arginine into citrulline (deimination). Deimination associates with or is a signal for apoptosis [4], and antagonizes histone arginine methylation contributing to transcription control [5], [6]. More, PADs are though to be key factors in the pathophysiology of multiple sclerosis, Alzheimer's disease, and rheumatoid arthritis [7], and in the metabolism of the major epidermal barrier protein filaggrin. Filaggrin (Unigene Hs.654510) is only expressed in the most differentiated keratinocytes of the epidermis within the so-called granular layer. Filaggrin associates with keratin intermediate filaments and facilitates the formation of the intracellular fibrous matrix during the transition from keratinocytes to corneocytes, which constitutes the cornified layer of the epidermis. Filaggrin is extensively deiminated at multiple residues in the early corneocyte [8]. Because of the lower affinity of deiminated filaggrin for keratins, it was proposed that filaggrin deimination facilitates its dissociation from the matrix and subsequent proteolysis [8]. Proteolysis of the deiminated filaggrin yields to a heterogeneous pool of aminoacids which contributes to the Natural Moisturizing Factor (NMF) [9]. The critical role of filaggrin in epidermis homeostasis and barrier function has been evidenced by the recent disclosure that loss-of-function mutations in the filaggrin gene underlie ichthyosis vulgaris (OMIM #146700) and are strong predisposing factors for atopic dermatitis (OMIM %603165)[10]. Abnormal filaggrin deimination should result in altered function and/or turn-over; with possible deleterious consequences in epidermis barrier function which might contribute to the outcome of atopy or other disorders. PAD3 is a strong candidate for filaggrin deimination. Among the *PADI* genes, only *PADI*3 shows specific expression in the granular layer and co-localization with filaggrin in the cytosol of granular keratinocytes and in the matrix of the lower corneocytes [11]. The enzymatic properties of PAD3 and its ability to deiminate filaggrin *in vitro* [12] further support the assumption that PAD3 is the best candidate for filaggrin deimination *in vivo*. In the search for new regulators of filaggrin deimination, we investigated the control of the genes *PADI*. We have reported that the *PADI*3 minimal promoter region showed a significant, albeit moderate, increase in activity (1.5-fold) in differentiated versus proliferative keratinocytes *in vitro*, and required the bound transcription factors NF-Y and Sp1/3 [13]. However, such co-operation is not specific to differentiated keratinocytes since NF-Y and Sp1 govern the transcription of a variety of genes in epithelial or non epithelial cells [14]–[17], as that of the ΔNp63 isoform of p63, a homolog of the p53 tumour suppressor gene, in proliferative mouse keratinocytes [18]. In the cervical carcinoma HeLa cells, NF-Y and Sp1 enhanced the levels of transcription of the topoisomerase II α and β gene promoters [19], [20] whereas the *PADI*3 gene promoter is not active [13].

By a comparative genomic approach, we have identified between *PADI*2 and *PADI*1 an 8-kb region (IG1) which clusters 19 conserved non coding segments (CNS) [3]. We here disclose that IG1 contains a calcium responsive enhancer which dramatically triggers the activity of the *PADI*3 gene promoter located 86-kb from it upon epidermal keratinocyte differentiation, and links *PADI*3 expression to AP-1 transcription factors through chromatin opening and looping.

## Methods

### Cell culture

All human samples were obtained after informed consent according to Helsinki principles. Normal Human Epidermal Keratinocytes (NHEKs) isolated from healthy foreskin and the A431 and HaCaT lines (a gift from N. Fusenig, German Cancer Research Center, Heidelberg, Germany) were cultured in a serum-free growth medium (KGM2, Promocell, Heidelberg, Germany) in the presence of 13 mg/ml bovine pituitary extract, 0.125 ng/ml Epidermal Growth Factor, 5 µg/ml insulin, 0.33 µg/ml hydrocortisone, 10 µg/ml transferrin, 0.39 µg/ml epinephrin, and with either 0.2 mM or 1.5 mM Ca2+. Human dermal fibroblasts and HeLa cells were maintained in DMEM with 10% (v/v) fetal bovine serum (Invitrogen, Carlsbad, CA).

### Genomic segments and plasmids

All sequences coordinates refer to a previously deposited sequence (Genbank accession number AJ549502). The primers used in this work are listed in supplementary Table S1. The promoter regions of the genes *PADI*1-4 comprise the proximal promoters published elsewhere [13], [21]–[23]. Genomic fragments were generated by PCR and subcloned into the pGL3 vector (Promega, Madison, WI). Site-directed mutagenesis was performed using the Vent DNA polymerase and *Dpn*I (New England Biolabs, Ipswich, MA). All constructs were checked by sequence analysis. Dominant-negative plasmids were generous gifts from Melanie Cobb (ERK1-2 mutants), Michael Karin (h-ras, h-raf constructs), and Michael Birrer (TAM67 c-Jun mutant). The plasmid pAP1-luc (BD Bioscience, Mountain View, CA) was kindly provided by Franck Verrechia.

### Transient transfections and luciferase assays

Actively proliferating NHEKs were seeded at a density of 25 000 cells/cm^2^ 24 hours before they were transfected by 1 µg total DNA using the TransFast reagent (Promega). The plasmid pCR2.1 (Invitrogen) was used to adjust for the mass of the input DNA when necessary. The plasmid pRL-TK (Promega), used to correct for transfection efficiency, was used at a 1/100 mass ratio versus the pGL3 plasmids. After transfection, the NHEKs were incubated 72 hours in the presence of either 0.2 mM or 1.5 mM calcium before lysis and dual luciferase assay (Promega). All assays were done in triplicate wells, and the experiments were repeated at least two times. Statistical analysis was performed using the StatEL software (Adscience, Paris, France).

### Real time quantitative (Q-)PCR analysis

All Q-PCRs were based on a SyBr-green based PCR mixture (MP Biomedicals, Solon, OH) and an ABI7300 system (Applied Biosystems, Foster City, CA). All primers pairs were designed using the Primer3 online software (http://frodo.wi.mit.edu/) and characterized by real-time amplification of a series of dilution of naked genomic DNA to determine linearity range and primer efficiency. All Q-PCR amplifications were done in triplicate, and the experiments were performed at least twice.

### DNase I hypersensitivity assay

We queried the DNase I hypersensitivity of genomic segments as devised elsewhere [24], using oligonucleotides probes listed in supplementary Table S1.

### Electrophoretic Mobility Shift Assay (EMSA)

Nuclear proteins were extracted as devised elsewhere [25], and protein amounts were quantified using a Bradford assay (Amresco, Solon, OH). EMSA was performed as reported elsewhere [26]. Briefly, 5 µg of whole cell extract from differentiating NHEKs was incubated in a binding buffer containing 10 mM Hepes, pH 7.8, 50 mM KCl, 2 mM dithiothreitol, 1 mM EDTA, 5 mM MgCl_2_, 10% glycerol, 3 mM 4-(2-aminoethyl)benzenesulfonyl fluoride, 2 mg/ml poly(dI-dC), and 2 mg/ml bovine serum albumin in a total volume of 20 µl at 25 °C for 20 min in the presence of 20 fmol of biotin-end-labeled double-strand oligonucleotide probe. DNA-protein complexes were resolved by electrophoresis on 4–6% polyacrylamide gels in 0.5×TBE buffer (50 mM Tris, 45 mM Boric Acid, and 0.5 mM EDTA). Five or 10 µg of antisera against c-Jun (BD, Franklin Lakes, NJ), JunB, JunD, c-Fos, FosB, Fra1 and Fra2 (Santa Cruz Biotechnologies, Santa Cruz, CA) were used in supershift assays. Nucleoprotein complexes were revealed by the Light Shift chemoluminescent assay kit (Pierce, Rockford, IL). Oligonucleotide probes were designed on the basis of the sequence of the centromeric and telomeric AP-1 sites of PIE. Competitors were unlabelled oligonucleotides with the identical sequence (wild-type competitor), or unlabelled oligonucleotides in which the AP-1 consensus sites were mutated (mutated competitor). All probe and competitor sequences are detailed in supplementary Table S1.

### Chromatin ImmunoPrecipitation (ChIP) assay

ChIP was carried out as published elsewhere [27] using 20×10^6^ NHEKs per experiment. Twenty-five µg of pre-cleared chromatin were immunoprecipitated with 5 µg of the same antibodies that described above, or 5 µg of a monoclonal antibody against the HA epitope (Millipore, Billerica, MA). One tenth of the immunoprecipitated DNA samples and 5 ng input DNA samples were submitted to Q-PCR.

### Chromosome Conformation Capture (3C)

Detailed protocol for 3C in NHEKs is provided as Supplementary Information S1.

Briefly, the 3C assay was performed as described previously [28] using *Bgl*II and *BamH*I for enzymatic digestion of chromatin, and primers designed towards the *BamH*I restriction sites. A sonication step was found to be necessary after formaldehyde cross-linking of NHEKs for efficient cell lysis and nuclei release, as verified by microscopic examination, but had no detectable effect on chromatin size, as checked by agarose gel electrophoresis. Agarose gel electrophoresis of a purified aliquot of the chromatin restricted by *Bgl*II and *BamH*I showed no detectable difference in digestion efficiency of samples from unstimulated or calcium-stimulated keratinocytes. Quantitative amplification of a segment of the *PADI*3 gene promoter was used for data normalization. For controls, we used the Bacterial Artificial Chromosome (BAC, AL590644) which contains a 165-kb insert spanning the whole region from the intergenic sequence telomeric to the gene *PADI*2 to the first intron of the gene *PADI*4, thereby including IG1 and the genes *PADI*1 and 3. The BAC clone was used to generate the control for random association events: 1.5 ng of the BAC preparation, restricted and ligated as the nuclei samples, was used as a template in control amplifications. Primer pairs efficiency values were determined from amplifications of a series of dilutions of a control template in which all possible *BamH*I/*Bgl*II ligation products of the *PADI* gene locus should be equally represented, generated from 30 µg of the BAC preparation, restricted and ligated at a high concentration (300 ng/µl).

### RNA analysis

NHEKs were treated for 16 h by 20 µM NDGA (Sigma) in 0.1% DMSO (Sigma), or by 0.1% DMSO. Total RNAs purified using the RNeasy Protect kit (Qiagen) were used as a template for Im-PromII reverse transcriptase (Promega). Q-PCR analysis was performed according to the 2^−ΔCt^ method [29], using β2 microglobulin as the reference, PAD1-3 and involucrin as the targets, and untreated NHEKs as the calibrator. Standard deviation were calculated as advised [29] using the methods of standard propagation of error. Actual inhibition of the AP-1 activation system by NDGA was also checked by transfection experiments using the plasmid pAP1-luc.

## Results

**Figure 1:**
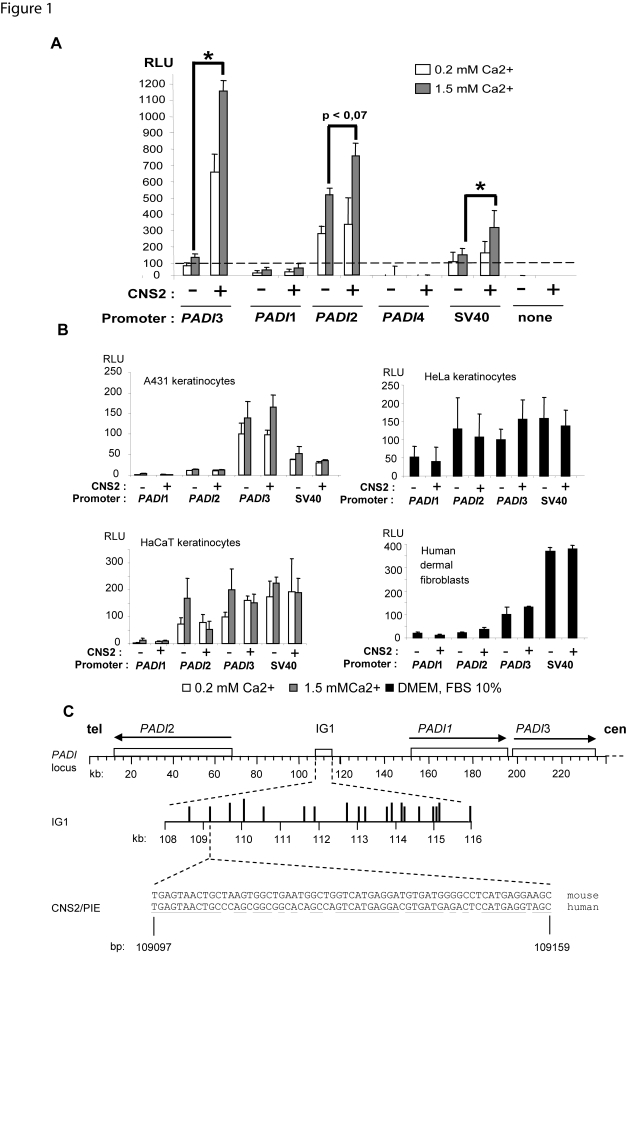
Identification of a strong transcriptional enhancer of the gene *PADI*3 in differentiated keratinocytes. A) The CNS2 comprises an enhancer of *PADI*3 gene promoter activity in differentiating NHEKs. NHEKs were transfected with luciferase reporter constructs containing the indicated promoter segment with or without the CNS2 which overlaps PIE, in the presence of 0.2 or 1.5 mM calcium. Values are expressed in Relative Luciferase Units (RLU), as means+/−SEM for at least 3 independent experiments, each performed in triplicate wells.*, p<0.05. B) The CNS2 does not show any detectable enhancer activity either in A431 skin squamous cell carcinoma keratinocytes (top left), in HeLa cervix adenocarcinoma cells (top right), in immortalized HaCaT (bottom left) keratinocytes, or in human primary dermal fibroblasts (bottom right), or 3T3 transformed murine fibroblasts (not shown). C) Schematic representation of the PADI gene locus showing CNS2/PIE (asterisk), IG1 (open box) and the genes PADI1-3 (open boxes with arrows indicating the orientation of transcription). The coordinates in AJ549502 are shown in kb or bp. The telomeric (tel) and centromeric (cen) ends of the locus are shown. The 19 CNSs within IG1 are depicted by vertical bars of lenght depending upon their level of interspecies conservation (for details, see [3]). The entire mouse and human sequences of CNS2/PIE are shown. Underlined are the conserved bases between mouse and human. The centromeric end of the locus (approximatively 100 kb) including the genes *PADI*4 and 6 is not shown due to size limitation.

Normal human epidermal keratinocytes (NHEKs) grown *in vitro* in the presence of 0.2 mM calcium are actively proliferating. Upon exposure to 1.5 mM calcium, they undergo a differentiation program which closely resembles that of the epidermis; this well documented system recapitulates the natural epidermal calcium gradient [30], [31]. Consistently, in our hands, quantitative RT-PCR and/or western blot analyses show increased mRNA and/or protein levels of markers of the keratinocyte differentiation such as loricrin, involucrin, and the α-macroglobulin like inhibitor of protease A2ML1 (not shown, and [32]). The 19 CNS within IG1 are candidates for a role in transcription regulation because inter-species conservation of DNA segments is often predictive of a biological role. We designed a screening strategy based on transfection in NHEKs cultured in both conditions of luciferase reporter plasmids containing the promoter region of the gene *PADI*3, flanked by each of the 19 CNS. We observed a significantly increased activity of the *PADI*3 promoter region in the presence of the CNS2 (63 bp, nt 109097–109159). The effect was higher in calcium-stimulated (10-fold induction) as compared to unstimulated cells (6-fold induction) (Fig. 1A). None of the 18 other CNS showed significant and reproductible effect upon the *PADI*3 gene promoter activity. The activity of the CNS2 did not depend on its orientation (data not shown). This activity was not donor dependent, since it was observed in NHEKs from 4 unrelated individuals. We observed a moderate enhancer effect on the *PADI*2 gene and the SV40 virus promoter regions in the presence of 1.5 mM calcium. We next queried wether such enhancer activity could be detected in keratinocyte lines. Unexpectedly, the activity of the CNS2 could not be detected in HaCaT immortalized keratinocyte though they are able to differentiate *in vitro* upon calcium exposure (Fig. 1B). We assume that the genetic alterations present in the HaCaT lineage (one single copy of chromosome 3p, 4p, and 9p and an additional copy of 9q at least) impair the cell responsiveness to CNS2 directly or not. However, the underlying mechanism remains undetermined, given the complexity of genetic alterations and accumulating changes upon passaging in such immortalized cells.

No effect of CNS2 was observed in skin squamous cell carcinoma keratinocytes A431 and cervix adenocarcinoma HeLa cells which are unable to differentiate *in vitro* (Fig. 1B). Neither was it detected in primary human dermal fibroblasts (Fig. 1B), nor 3T3 transformed murine fibroblasts (not shown). These results suggested sensitivity to cell type and status.

**Figure 2:**
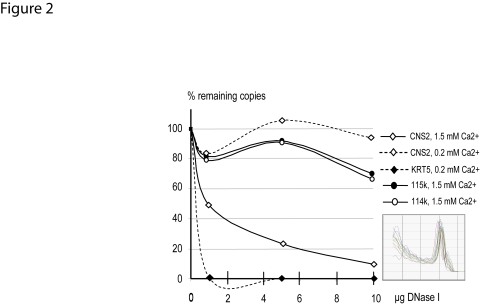
Hypersensitivity of the CNS2 to DNase I. The DNase I-digestion profiles of the CNS2 including PIE, the *KRT*5 gene promoter region, and the two segments 114k (a LINE repeat, nt 114-549-114655) and 115k (nt 115764–115778) located at the centromeric end of IG1 are shown. The amount of template DNA was standardized by correcting for amplification of the *HBE*1 promoter region sequence. Inset: representative dissociation curves.

Because active enhancers are known to be located in open chromatin regions and thereby show DNase I hypersensitivity, we used a real-time PCR-based approach to assess the integrity of the CNS2 in DNase I treated nuclei prepared from NHEKs [24]. This method had been reported efficient to query the nuclease sensitivity of candidate segments [33], [34] or to build comprehensive maps of open chromatin sites at the genomic or chromosomic levels [35], [36]. We normalized the number of intact remaining copies of CNS2 or of other probes to that of a segment of the promoter region of the fetal ε-globin gene (*HBE*1) which is not transcribed in NHEKs. No significant DNase I hypersensitivity of the CNS2 was detected in proliferating NHEKs, in which a fragment spanning the promoter of the keratin 5 gene, a strongly expressed marker of proliferative keratinocytes, showed hypersensitivity (Fig. 2). In contrast, in calcium-stimulated keratinocytes, increasing amounts of DNase I associated with a dramatic loss of amplifiable copies of the CNS2 (Fig. 2), supporting its role as an enhancer prone to bind transcription activators upon keratinocyte differentiation. Two segments located at the centromeric ends of IG1 showed no hypersensitivity to DNase I (Fig. 2), suggesting that the increased sensitivity to DNAse I we observed was due to local alteration in chromatin accessibility in the close or distant vicinity of the CNS2, and not to a chromatin opening event at the chromosome scale. We concluded that the CNS2 overlaps a strong calcium-dependent enhancer of the *PADI*3 gene in NHEKs. Therefore we renamed it Peptidylarginine deiminase Intergenic Enhancer (PIE, EU443247 and AJ549502).

**Figure 3:**
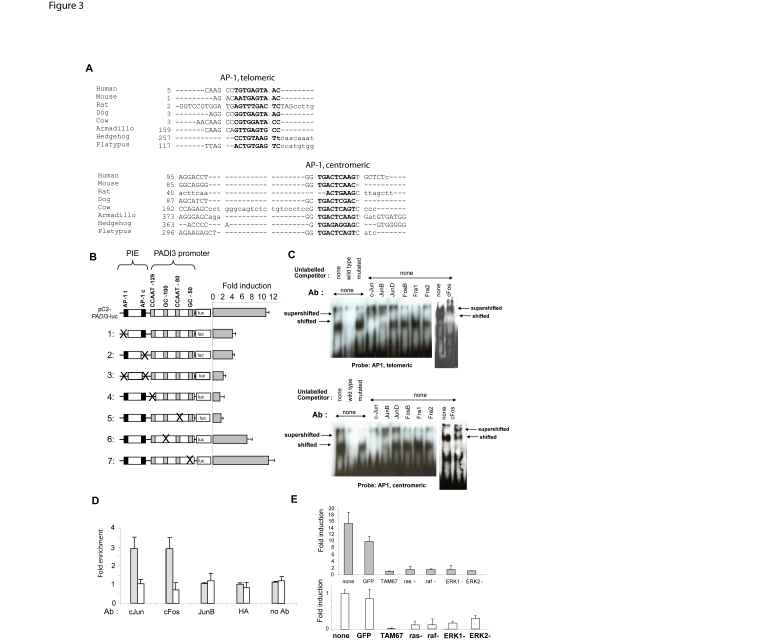
AP-1 factors binding and proximal CAAT motifs required for enhancer activity. A) Two conserved AP-1 sites were identified in PIE. Alignment of the sequences of the conserved telomeric or centromeric AP-1 putative binding sites (bold) in PIE in human, mouse, rat, dog, cow, armadillo (*Dasypus novemcinctus*), hedgehog (*Echinops telfairi*) and platypus (*Ornithorhynchus anatinus*). Sequence retrieval and analyses were performed using the Ensembl web site (http://www.ensembl.org), the Vista suite (http://genome.lbl.gov/vista/index.shtml), and the program Dialign-TF (http://www.genomatix.de) with default parameters. Note that the centromeric site is more highly conserved that the telomeric site. B) AP-1 and CAAT recognition motifs are mandatory for enhancer activity. Luciferase (luc) plasmids were engineered with invalidated telomeric (AP-1t) and centromeric (AP-1c) AP-1 sites in PIE (lines 1–3), or invalidated CCAAT or GC boxes in the *PADI*3 promoter (cross) (lines 4–7). Line pC2-*PADI*3-luc: shown is the ratio between the luciferase activity of the *PADI*3 promoter segment with PIE versus that without PIE. Lines 1–3: shown are the ratios between the luciferase activities of the mutated constructs versus that of the construct containing the *PADI*3 promoter segment without PIE. Lines 4–7: shown are the ratios between the luciferase activities of the mutated constructs with PIE versus that of their counterparts without PIE. Values are expressed as means+/−SEM for 2 independent experiments, each performed in triplicate wells. C, D) Bound c-Fos/c-Jun are required for PIE function. Electrophoretic mobility shift assays (C) showed that both telomeric (AP-1t) and centromeric (AP-1c) AP-1 sites are expressed and able to bind nuclear factors from differentiating NHEKs, generating a shifted band, which is no longer detected in the presence of unlabeled wild type competitor. When antibodies specific to c-Jun, JunB or c-Fos were used, the shifted band was either supershifted or absent due to the formation of antibody-containing higher molecular weight complexes which do not enter the gel. The binding of c-Jun and c-Fos to PIE in living NHEKs was confirmed by chromatin immunoprecipitation (D). Immunoprecipitated chromatin from NHEKs grown in the presence of 1.5 mM calcium showed about 3-fold relative enrichment in c-Jun or c-Fos bound to PIE as compared to that from NHEKs grown in the presence of 0.2 mM calcium (grey bars). No enrichment was observed using antibodies to JunB or the irrelevant anti-hemaglutinin (HA), nor when we queried the unrelated CNS3 which is centromeric to the CNS2 (nt 109913–109973) (white bars). Data are shown as means+/−SEM for 2 independent experiments performed in triplicate wells. E) Blocking c-Jun or the ras/raf/ERK cascade impairs the activity of PIE. Differentiating NHEKs were co-transfected with reporter plasmids containing the *PADI*3 gene minimal promoter region fitted or not with PIE, and an expression vector coding for dominant negative mutants of c-Jun (TAM67), ras (ras-), raf (raf-), ERK1 and 2 (ERK1- and ERK2-, respectively) or the GFP as a control (GFP) (top). For these co-transfections, the luciferase activities generated by the plasmids containing PIE were compared with that generated by the plasmids containing only the *PADI*3 gene promoter. Parallel co-transfection experiments using the plasmid pAP1-luc showed dramatic loss of AP-1 activity in NHEK transfected with the dominant negative mutants (bottom). For these co-transfections, the luciferase activities generated by pAP1-luc co-tranfected with mutants were compared with that generated by pAP1-luc transfected alone. The plasmid pCR2.1 was used to adjust for the mass of the input DNA. Values are expressed as means+/−SEM for 2 independent experiments performed in triplicate wells.

BLAST searches in the most recently available mammalian and nonmammalian draft genomes of the Ensembl database and the trace archives of the National Center for Biotechnology Information (NCBI), followed by sequence analysis identified one highly conserved canonical AP-1 binding sequence at each ends of PIE (Fig. 3 A). Invalidation of one or another or both AP-1 sites by site-directed mutagenesis resulted in the almost complete loss of PIE enhancer activity in luciferase assays (Fig. 3 B, lanes 1–3). Similarly, deletion of one or another or both AP-1 sites within the reporter plasmids resulted in the loss of PIE activity (not shown). In the epidermis, the AP-1 family groups heterodimeric proteins consisting of members of the Jun (c-Jun, JunB, JunD), or Fos (c-Fos, FosB, Fra1, Fra2) families, or Jun homodimers. Electrophoretic mobility shift assays (EMSA) with or without antibodies specific to each member of the Jun/Fos families was performed using nuclear extracts from differentiating NHEKs to assess wether the binding of AP-1 factors could account for the observed activity of PIE in these cells. EMSA revealed that c-Jun, c-Fos and JunB were expressed and able to bind the two AP-1 sites within PIE in differentiating keratinocytes (Fig. 3 C). Chromatin immunoprecipitation (ChIP) assays revealed a significantly increased occupancy of PIE by c-Jun and c-Fos in stimulated versus unstimulated NHEKs (2.9 and 2.8 -fold, respectively) (Fig. 3 D, grey boxes). No significant increase in occupancy by c-Jun and c-Fos was found when we queried a non related, non coding segment within IG1 (CNS3) (Fig. 3D, white boxes). Last, the effect of PIE on the *PADI*3 gene promoter was found impaired in stimulated NHEKs co-transfected with the c-Jun dominant negative mutant TAM67 [37] (Fig. 3 E). We concluded that c-Jun and c-Fos are recruited at PIE and that c-Jun at least is mandatory for the enhancer activity of PIE in differentiating NHEKs. This is consistent with c-Jun and c-Fos being detected in the epidermal granular layer, c-Jun being specific of this later in most reports [38], [39]. Since the basal activity of the *PADI*3 core promoter required binding of the factors Sp1/3 and NF-Y [13], we mutated the CCAAT or the GC-boxes, which bind NF-Y and Sp1/3 respectively, within the *PADI*3 promoter sequences in reporter plasmids. When inserted upstream the promoter sequence with any of the CCAAT boxes invalidated, PIE no longer showed any effect on luciferase activity. In contrast, mutations invalidating the GC boxes did not impair PIE enhancer effect (Fig. 3 B, lanes 4–7). These results suggested functional interaction of PIE with the CAAT boxes, presumably through c-Jun interacting with NF-Y as shown for the p21(WAF1/Cip1) gene [40]. We failed to detect by ChIP significant amounts of c-Jun at the promoter level (not shown). We assume this was due to the limits of ChIP when applied to proteins associated with chromatin without being directly bound to the DNA helix [41].

AP-1 activity has been shown to be induced by the Extracellular-Regulated Kinase (ERK) signalling pathway upon keratinocyte differentiation [42]. Cotransfections of NHEKs with the PIE-*PADI*3 promoter reporter plasmid and any of the dominant negative mutants of ras, raf, ERK-1 or -2 all resulted in a dramatically decreased gain of the *PADI*3 gene promoter activity (Fig. 3 E), indicating that PIE is a downstream effector of AP-1 through the ras/raf/ERK cascade. The expression of the dominant negative mutants associated with the loss of AP-1 activity, as controlled by parallel co-transfection experiments using the plasmid pAP1-luc designed for direct monitoring of the activity of the AP-1 activation pathway (Fig. 3 E).

**Figure 4:**
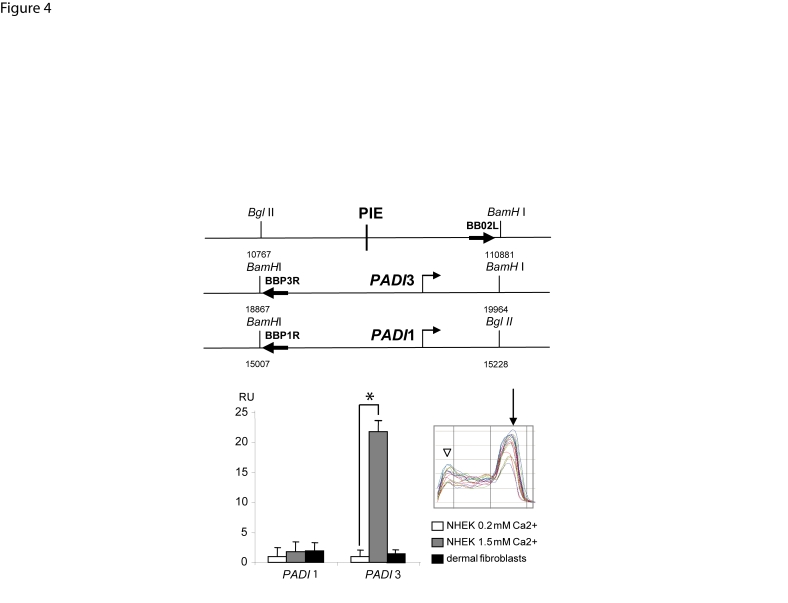
Increased frequency of interaction between PIE and the *PADI*3 promoter indicating chromatin looping. The abundancy of chimeric DNA containing PIE and the promoter region of the genes *PADI*1 or *PADI*3 were explored by 3C analysis using specific primers (top). The levels of PIE-*PADI*3 promoter chimeric DNA in extracts from NHEKs grown in the presence of 1.5 mM calcium were approximately 20-fold greater than that in NHEKs grown in the presence of 0.2 mM calcium, or dermal fibroblasts. Amplification of BAC-based negative control for random association events (see methods) yielded no product (not shown). All amplifications were performed in triplicate wells. Results were normalized with respect to the abundancy of the *PADI*3 promoter sequence in the template. Data are expressed in Relative Units (RU) as means+/−SEM for 2 independent experiments, each performed in triplicate wells.*, p<0.05. Representative dissociation curves (inset) show the major amplimer peaks (arrow) along with secundary peaks (arrowhead) indicative of primer-dimer formation in some experiments. Such possible primer-dimer was not detectable in ethidium-bromide stained agarose gel of the end-point PCR reactions and did not seem to interfere with calibration curves.

Recent evidence showed close proximity between long-range enhancers and promoters through chromatin loops [43]. We searched for non-random alterations in the frequency of contacts between the *PADI*3 gene promoter and PIE in living keratinocytes by the use of Chromosome Conformation Capture (3C, Fig. 4) [44]. Controls were included as advised elsewhere [45] (see methods). Chimeric DNA containing PIE and the *PADI*1 or *PADI*3 promoter regions were amplified using the oligonucleotide pairs BB02L/BP1R and BB02L/BP3R, respectively (Fig. 4, top). 3C allowed detection of chimeric DNA containing PIE and the *PADI*3 promoter region, generated by self-ligation of physically close fragments from crosslinked and restricted chromatin. More, the abundancy of such PIE-*PADI*3 chimeric DNA was found to be more than 20-fold greater in stimulated keratinocytes than that in unstimulated keratinocytes or dermal fibroblasts (Fig. 4, bottom), a magnitude greater than that reported for the β globin chromatin hub [28], [46]. In contrast, the ratio between the abundancy of PIE-*PADI*1 chimeric DNA in unstimulated versus stimulated NHEKs was found 9-fold lower than that corresponding to PIE and *PADI*3 promoter, and not sensitive to calcium-stimulation (Fig. 4). No chimeric DNA could be amplified from the BAC-based control for random association events (Fig. 4), or from crude genomic DNA (not shown). This indicated non random physical association between PIE and the *PADI*3 gene promoter in the nuclei of differentiating NHEKs, through a chromatin looping spanning 86-kb.

**Figure 5:**
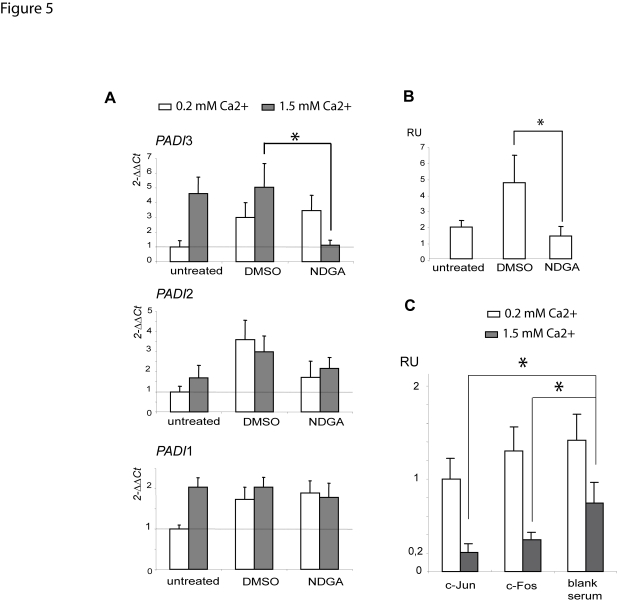
NDGA blocks calcium-induced increase of the *PADI*3 mRNA levels and impairs c-Jun or c-Fos recruitment at PIE. Quantitative RT-PCR analysis of the steady-state levels of *PADI*1-3 (A) and involucrin (B) mRNAs was performed using unstimulated or calcium-stimulated NHEKs grown in the presence of 20 µM NDGA in 0.1% DMSO, or 0.1% DMSO, and the β2-microglobulin mRNA as the reference. Data are expressed as means+/−SEM for 2 independent experiments, each performed in triplicate wells.*, p<0.05. A) NDGA blocks calcium-induced increase of the mRNA levels of *PADI*3, but not that of *PADI* 1, 2. Treatment with 0.1% DMSO had no effect on the *PADI*3 mRNA levels (2^(−ΔΔCt)^) in calcium-stimulated NHEKs. Treatment with 20 µM NDGA resulted in a dramatic decrease of *PADI*3 mRNA levels in calcium-stimulated NHEKs. B) NDGA blocks calcium-induced increase of the involucrin mRNA levels. In untreated NHEKs, calcium stimulation resulted in a two fold increase in involucrin mRNA levels. In NHEKs treated with the carrier (DMSO), calcium stimulation associated with a five fold increase in involucrin mRNA levels. In NHEKs treated with NDGA, calcium stimulation associated with only a 1.5 fold increase in involucrin mRNA levels. Together these results suggested that treatment with NDGA blocked calcium-induced increase of the *PADI*3 and involucrin mRNA levels. RU, relative units. C) NDGA impairs c-Jun or c-Fos recruitment at PIE in calcium-stimulated NHEKs. ChIP analysis of PIE occupancy by c-Jun/c-Fos was performed in the presence of NDGA or the carrier (DMSO). Immunoprecipitated chromatin from NHEKs grown in the presence of 1.5 mM calcium showed strongly decreased occupancy of PIE by c-Jun (20% remaining) or c-Fos (34% remaining) but not non specific factors (74% remaining) in the presence of NDGA as compared to the same cells grown in the presence of the carrier. Immunoprecipitated chromatin from NHEKs grown in the presence of 0.2 mM calcium showed no decrease in the occupancy of PIE by c-Jun (99% remaining) or c-Fos (130% remaining) or non specific factors (140% remaining) in the presence of NDGA as compared to NHEKs grown in the presence of the carrier. Data are expressed as Relative Units (RU) and shown as means+/−SEM for 2 independent experiments performed in triplicate wells. *, p<0.05.

We next queried whether the inhibition of the AP-1 pathway could have an effect upon the *PADI*3 mRNA levels in differentiating NHEKs (Fig. 5). We used nordihydroguaiaretic acid (NDGA), a natural polyphenolic compound reported to inhibit c-fos mRNA and protein levels, as well as binding of the fos-jun dimer to DNA in keratinocytes [47], [48] and *in vitro* assays [49]. It also inhibits the auto-regulated synthesis of c-jun mRNA in TPA-stimulated HL60 cells [50]. Consistently, transfection of pAP1-luc yielded no significant luciferase activity in keratinocytes treated with NDGA, in contrast to keratinocytes untreated or treated by the carrier (0.1% DMSO) (not shown). We performed real-time RT-PCR to investigate the levels of mRNA in calcium stimulated or unstimulated keratinocytes, treated or not with NDGA. For unknown reasons, 0.1% DMSO resulted in an increase of the levels of the *PADI*3 transcripts in unstimulated NHEKs, but had no effect in stimulated NHEKs (Fig. 5A). Whatsoever, treatment with NDGA resulted in a dramatic decrease of *PADI*3 mRNA levels in stimulated NHEKs, to a level close to that observed in untreated unstimulated NHEKs (Fig. 5A). We found no sensitivity to NDGA treatment of the *PADI*1 or *PADI*2 mRNA levels in calcium-stimulated NHEKs (Fig. 5A). We next queried the effect of NDGA upon the mRNA levels of a well-established AP-1 target gene, the early marker of keratinocyte differentiation involucrin [51](UniGene Hs.516439) (Fig. 5B). In untreated NHEKs, calcium stimulation resulted in a two fold increase in involucrin mRNA levels. In NHEKs treated with the carrier (DMSO), calcium stimulation associated with a five fold increase in involucrin mRNA levels. In NHEKs treated with NDGA, calcium stimulation associated with only a 1.5 fold increase in involucrin mRNA levels. Together these results suggested that treatment with NDGA blocked calcium-induced increase of the *PADI*3 and involucrin mRNA levels and were consistent with the assumption that targeting the AP-1 system by NDGA has a negative effect upon *PADI3* expression. We next performed ChIP analysis to investigate the influence of NDGA upon PIE occupancy by c-Jun/c-Fos (Fig. 5C). We compared the number of copies of PIE present in fractions immunoprecipitated with the c-Jun antiserum or the c-Fos antiserum or a blank rabbit serum, from NHEKs grown in the presence of 0.2 mM or 1.5 mM calcium with or without NDGA. Immunoprecipitated chromatin from differentiating NHEKs grown in the presence of NDGA revealed strongly decreased occupancy of PIE by c-Jun (20% copies remaining) or c-Fos (34% copies remaining) as compared to differentiating NHEKs grown in the presence of the carrier (Fig. 5C). Immunoprecipitated chromatin from differentiating NHEKs grown in the presence of NDGA showed a much lower decrease in the non specific occupancy of PIE (74% remaining) as compared to the same cells grown in the presence of the carrier (Fig. 5C). Immunoprecipitated chromatin from NHEKs grown in the presence of 0.2 mM calcium showed no decrease in the occupancy of PIE by c-Jun (99% remaining) or c-Fos (130% remaining) or non specific factors (140% remaining) in the presence of NDGA as compared to the same cells in the presence of the carrier (Fig. 5C). These results strongly suggested that NDGA treatment is associated with a dramatic decrease in c-Jun or c-Fos recruitment at PIE. Altogether these findings supported that proper AP-1 activation system and PIE occupancy by c-Jun or c-Fos are necessary for calcium-dependent increase of the *PADI*3 mRNA levels.

## Discussion

**Figure 6:**
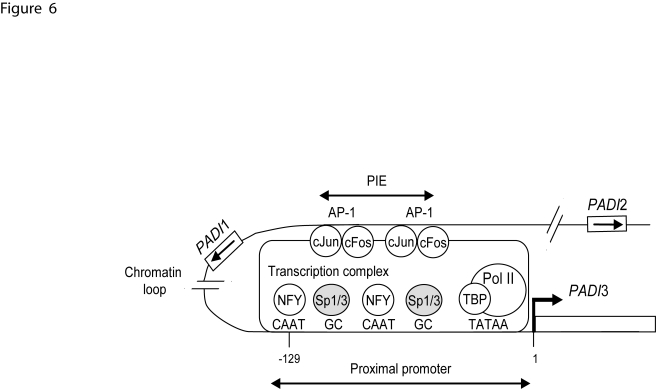
Possible model for *PADI*3 promoter regulation. The binding of cJun and/or cFos dimers to the AP-1 sites of PIE is enhanced in calcium-stimulated keratinocytes and increases the rate of transcription of *PADI*3 (broken arrow). Transcription factors with functional association (cJun, cFos, NF-Y) are shown as open circles. Sp1/3 factors are denoted as grey circles. Transcription factors binding sites are indicated (capital letters). The arrows show the transcription orientation of the genes *PADI*1-2 (white boxes). TBP: TATA-box binding protein; pol II: RNA polymerase II.

Our findings show that a distal enhancer located 86-kb telomeric to the gene *PADI*3, namely PIE, underlies efficient transcription of this gene in calcium differentiated epidermal keratinocytes through binding to AP-1 factors and in association with chromatin looping (Fig. 6). To our knowledge, PIE exhibits all requirements for an enhancer: its sequence is highly conserved in mammals, it exerts a strong effect on the *PADI*3 gene promoter *in vitro*, and, in conditions when the gene *PADI*3 is specifically upregulated, it is DNase I hypersensitive, shows non random physical proximity to the *PADI*3 gene promoter, and binds transcription activators (AP-1). We found that the AP-1 inhibitor NDGA suppresses the calcium-induced increase of *PADI*3 mRNA levels in keratinocytes, and the occupancy of PIE by c-Jun and c-Fos. However, possible non-specific cellular effects of NDGA can not be ruled out. Targeting experiments with silencing RNAs specific to c-Jun and/or c-Fos would help to adress this issue.

We observed an effect of PIE upon the heterologous SV40 promoter, such as observed with a variety of other AP-1 bound distal enhancers as those for the human renin gene promoter [52], the mouse laminin α3A gene [26], or the sheep interferon τ gene [53]. It is likely due to the positive interaction between c-Jun and the factor Sp1 which binds to the SV40 promoter, as observed in a number of studies [54]–[56]. Direct interaction with the viral TATA-box throught the TATA-box binding protein (TBP) is also a possible mecanism, given the ability of c-Fos and c-Jun proteins to interact directly with TBP [57].

Because PIE is located within the *PADI*1-2 intergenic region, it supports the assumption that the use of distant *cis*-regulatory elements contributes to maintain the physical organization of the locus [3]. We retrieved ortholog PIE sequences from placental mammals and monotreme (platypus) (Fig. 3A), but not from nonmammalian vertebrates, what suggests that PIE is a mammalian novelty. The role of the other CNS in IG1, should they have any, is unknown. It is possible that they exert a function respective to other *PADI* genes, cell types, or stimuli than the ones we queried. We recently identified another calcium-responsive enhancer activity lying within a large restriction fragment of IG1 (VA, unpublished data), presumably arising from the possible co-operation between two or more CNS with no detectable activity when assayed individually, or from a subsequence that had not been listed as a CNS because of a lower degree of conservation.

AP-1 factors are key regulators of cell life and death through the control of gene transcription in response to extracellular stimuli such as growth factors or cytokines [58]. Specifically, c-Jun/c-Fos heterodimers have been reported to be activators of the transcription of the genes encoding profilaggrin [59], loricrin, involucrin and transglutaminase 1 [60], all critical components of the terminally differentiated keratinocyte. Also, our results pave the way to the exploration of deimination control during tumorigenesis and wound healing, two conditions for which AP-1 factors are critical [61], [62]. Our results reveal that the ras/raf/ERK cascade is required for PIE activity during calcium-mediated NHEK differentiation. However, AP-1 factors can also be activated by the JNK/P38 kinase pathway upon stimulation by pro-inflammatory cytokines or genotoxic stress [58], and AP-1 transactivation is a key downstream events that results from UVB irradiation in keratinocytes [63]. Whether the JNK/P38 pathway has a role on PIE activity remains to be addressed, and would be of interest with respect to the molecular responses of epidermal keratinocytes to genotoxic stress.

This is the first time distant regulation of a *PADI* gene is reported. We observed local chromatin opening and chromatin looping in conditions when PIE-bound AP-1 factors trigger transcription of the gene *PADI*3. Though we can not rule out that these events are simply correlative, together our findings strongly support that the increased proximity between PIE and the *PADI*3 gene promoter through chromatin bending is at least a major contribution in *PADI*3 enhanced transcription. This is consistent with the model that chromatin plasticity is a key feature for genome expression [2], [44]. Long-range enhancers have been identified in a variety of chromosomal regions and tissues, at a distance up to 1-Mb from their cognate promoters [2]. Chromatin loops have been reported to play a role in the cell specificity of gene expression, in the control of transcriptional noise level, and in the activation of cooperative interactions [64]. PIE is located 86-kb away from the *PADI*3 gene promoter on chromosome 1p36. It has been proposed that the Wilms' tumour suppressor gene, *WT*1, also located on 1p35-36, is regulated by an enhancer away from more than 9-kb of its 5′ proximal region [65]. The identification of a distant transcriptional enhancer of the gene *SPRR*1A within the 5-Mb epidermal differentiation complex (EDC) on 1q21 was an important finding to understand the control of its expression in keratinocytes *in vitro* and *in vivo* [66]. More, the transcription of the p63 gene is regulated by a keratinocyte-specific enhancer located 160-kb away on chromosome 3q28 [67]. Together with these studies, our findings support that transcriptional regulation at a distance might be a key issue in keratinocyte biology.

In conclusion, the genetic bases of the distinctive patterns of expression of the genes *PADI* are unclear, despite the characterization of their respective core promoters. We assume that long range control of transcription, involving IG1 or introns, should have a role in the regulation of deimination. A forthcoming transgenic mouse model will definitely elucidate the role of PIE *in vivo*, in normal or pathological conditions. The impairment of long range control underlies a variety of severe human disorders [2]. Therefore PIE and other CNS within IG1 can be reasonably considered as candidates in association studies for deimination-related disorders or atopic disease, and we have included them in a recently initiated screen of the *PADI* locus in the search for variant haplotypes possibly associated with rheumatoid arthritis.

## Supporting Information

Table S1 Oligonucleotide probes used in this study
(0.07 MB DOC)

Supplementary Information S1 3C detailed protocol for NHEKs
(0.03 MB DOC)
