# Supplementary material for: Long-Range Enhancer Associated with Chromatin Looping Allows AP-1 Regulation of the Peptidylarginine Deiminase 3 Gene in Differentiated Keratinocyte
Source: PLoS One. 2008 Oct 16;3(10):e3408. doi: 10.1371/journal.pone.0003408 (PMC2566589; doi:10.1371/journal.pone.0003408)
Supplement: Table S1 — Oligonucleotide probes used in this study (0.07 MB DOC) [file pone.0003408.s001.doc]

**Supplementary table S1. Oligonucleotide primers used in this study.**

Restriction sites are underlined (GCTAGC: *Nhe*I, GAGCTC: *Xho*I, AAGCTT: *Hind*III). AP-1 sites are in bold. Mutated bases are in lower-case.

| application | locus/gene | target | Forward (5'-3') | Reverse (5'-3') |
| --- | --- | --- | --- | --- |
| **amplification** |  |  |  |  |
| **of genomic segments** | PADI1 | promoter region | AGATCTGGTGAACCTGCGTGGAAG | AAGCTTCCTGTCACCTGGCTCCCA |
|  | PADI2 | promoter region | AGATCTAGAATGACCCATTTCACGAGC | AAGCTTCCTCCCCGCCGCAGT |
|  | PADI3 | promoter region | AGATCTCTTTCATGACTGGAGCCAATG | AAGCTTCATTGGCTCCAGTCATGAAAG |
|  | PADI4 | promoter region | AGATCTACCACTCTCGAGCTGTGGAAGAAC | AAGCTTCGTCGGGCTAGCTCGTCCCTCTG |
|  | IG1 | CNS2/PIE | GCTAGCACCAAGCCTGTGAGTAACTGC | GAGCTCGAGAGCACTTGAGTCACCA |
| **site directed mutagenesis** |  |  |  |  |
|  | PIE | AP-1-t | CACCAAGCC**TGaGtGaAtCT**GCCCAGCG | GTACCGAGCTCTTACGCGTGCTAG |
|  |  | AP-1-c | **GGgGtCgCtAGt**GCTCTCGAGATCTCAAcatgg | GTACCGAGCTCTTACGCGTGCTAG |
|  | PADI3 promoter | CAAT box -1 | GTCCCTTCCAGatatcCCTGAGCTCTCAGC | CCAGTATTTGTATACGAACCTGTT |
|  |  | CAAT box -2 | CATGACTGGAGatatcGAATTGGCACCC | AAAGCCCAGCCCCTTCCCAG |
|  |  | GC box -50 | CTGGGCTGGAGggatccGGCCAGGGGCTTT | GGTGCCAATTCATTGGCTCC |
|  |  | GC box -100 | AGCTCTGGGAAGggaCtccGCTTTCATGAC | GAGAGCTCAGGATTGGCTGG |
| **EMSA** |  |  |  |  |
|  |  | AP-1-t (wild type) | TACCAAGCC**TGTGAGTAACT**GCCCAG | CTGGGC**AGTTACTCACA**GGCTTGGTA |
|  |  | AP-1-t (mutated) | TACCAAGCC**TGTcGTtACT**GCCCAG | CTGGGC**AGTaACgACA**GGCTTGGTA |
|  |  | AP-1-c (wild type) | GGAGGACCTGGTGACTCAAGTGCTCTCG | CGAGAGCACTTGAGTCACCAGGTCCTCC |
|  |  | AP-1-c (mutated) | GGAGGACCTGGgGtCgCtAGTGCTCTCG | CGAGAGCACTaGcGaCcCCAGGTCCTCC |
| **All real-time PCR including** |  |  |  |  |
| **DNase I assay, ChIP, and 3C** |  |  |  |  |
|  | PIE | PIE | GCCTCTGTACCAAGCCTGTG | GAGTCACCAGGTCCTCCTCA |
|  | *PADI*3 | promoter region | GGGGCTTTTAAACCTCATCC | GCTGGTGTTGGACTTAGCTGT |
| **DNase I hypersensitivity** |  |  |  |  |
| **assays** | IG1 | LINE (114k) | TGCATGGATGTTTGAGAGGA | AGGACACCAGCCTAATGTGG |
|  |  | non repeat segment (115k) | AAGCCCAGCACTTTGAGAAA | CCACCACACCCAGCTAATTT |
|  | *KRT*5 | promoter region | GGAAGGGATAAAAAGGGGGCAT | GGAGCAAGAGAACCAGGCACT |
|  | *HBE*1 | promoter region | TGGGTCTCACGAGATCTGATGGTT | ATGGCCAGGAGAAGCAAGTCACAT |

| **3C** |  |  |  |  |
| --- | --- | --- | --- | --- |
|  |  | PIE-*PADI*1 hybrids (BBP1R) | AATTCTAGGATACGGTCTTGACC(BB02L) | GCTTGGTCCCAGGGTAACAG (BBP1R) |
|  |  | PIE-*PADI*3 hybrids (BBP3R) | AATTCTAGGATACGGTCTTGACC(BB02L) | CACTCGCTCACTGTGTGACCTBP3R) |
| **RNA analysis** |  |  |  |  |
| The oligonucleotide primers were published elsewhere (Foulquier, C., Sebbag, M., Clavel, C., Chapuy-Regaud, S., Al Badine, R., Mechin, M.C., Vincent, C., | | | | |
| Nachat, R., Yamada, M., Takahara, H., et al. (2007) Peptidyl arginine deiminase type 2 (PAD-2) and PAD-4 but not PAD-1, PAD-3, and PAD-6 are expressed | | | | |
| in rheumatoid arthritis synovium in close association with tissue inflammation. Arthritis Rheum, 56, 3541-3553.) | | | |  |
