# Supplementary material for: Long-Range Enhancer Associated with Chromatin Looping Allows AP-1 Regulation of the Peptidylarginine Deiminase 3 Gene in Differentiated Keratinocyte
Source: PLoS One. 2008 Oct 16;3(10):e3408. doi: 10.1371/journal.pone.0003408 (PMC2566589; doi:10.1371/journal.pone.0003408)
Supplement: Supplementary Information S1 — 3C detailed protocol for NHEKs (0.03 MB DOC) [file pone.0003408.s002.doc]

**Supplemental information S1.**

**3C detailed protocol for NHEKs**

The 3C assay was performed as described previously (20) using 20 × 10^6^ cells. To disrupt the cells, the samples were briefly sonicated using the ultrasonic processor. This step was found to be necessary for efficient cell lysis and nuclei release, as verified by microscopic examination, but had no detectable effect on chromatin size, as checked by agarose gel electrophoresis of a 10% aliquot of the lysate, which was purified beforehand as devised in the ChIP section. DNA concentration in the whole lysate was inferred from that of this processed sample, as measured by UV spectrophotometry. Nuclei containing 40 µg of DNA were resuspended in restriction enzyme buffer #2 (NEB), and incubated at 37°C overnight with the compatible restriction enzymes *Bgl*II (200 units) and *BamH*I (400 units). Gel electrophoresis of a purified aliquot of the restricted chromatin showed no detectable difference in digestion efficiency of samples from unstimulated or calcium-stimulated keratinocytes. Digested chromatin (2 µg) was ligated using 4,000 units of T4 DNA ligase (NEB) overnight at 16°C in a final volume of 0.8 ml. DNA was purified by Proteinase K digestion, phenol/chloroform extraction, and ethanol precipitation. Samples were dissolved in 10 mM Tris-HCl, pH 8.0 and the concentration was adjusted to 100 ng/µl. The ligation products were PCR-amplified using the primers BB02L, BP1R and BP3R shown in figure below and listed in the supplementary table. Chimeric DNA containing PIE and the *PADI*1 or *PADI*3 promoter regions were amplified using the oligonucleotide pairs BB02L/BP1R and BB02L/BP3R, respectively. The PCR products were verified by DNA sequencing. Quantitative amplification of a segment of the *PADI*3 gene promoter was used for data normalization. The Bacterial Artificial Chromosome (BAC, AL590644, BACPAC Resource Center, Children's Hospital Oakland Research Institute, Oakland, CA) contains a 165-kb insert spanning the whole region from the intergenic sequence telomeric to the gene *PADI*2 to the first intron of the gene *PADI*4, thereby including IG1 and the genes *PADI*1 and 3. The BAC clone was used as a control for random association events: 1.5 ng of the BAC preparation, restricted and ligated as the nuclei samples, was used as a template in control amplifications. To generate a control template in which all possible *BamH*I/*Bgl*II ligation products of the *PADI* gene locus should be equally represented 30 µg of the BAC were restricted and ligated at a high concentration (300 ng/µl). Primer pair efficiency values were determined from amplifications of a series of dilutions of this control template.
